# Proximity of Water Wells to Public Water Testing Facilities in Alberta Using Drive Times

**DOI:** 10.1177/11786302221137437

**Published:** 2022-11-14

**Authors:** Abraham Munene, David C. Hall

**Affiliations:** 1 Faculty of Nursing, University of Alberta, Edmonton, AB, Canada; 2 Faculty of Veterinary Medicine, University of Calgary, Calgary, AB, Canada

**Keywords:** Well water, testing, public health, access, service area analysis

## Abstract

Approximately 10% of Albertans rely on well water for domestic purposes. The responsibility of water testing and stewardship is left to private well owners. Few well water owners conduct routine testing of their well water supplies. Drive times to public water testing facilities may be an important factor limiting a well owner’s ability to conduct routine water testing. The objective of this study is to describe the proximity of water wells, using drive times, to public water testing facilities and describe the availability of facilities based on hours of operation. Using network analysis, we determined the proportion of a sample of wells within 3 estimated drive times of public water testing facilities. 5872 wells were included in the sample. One hundred and seven water testing facilities were mapped within the province. Of the 5872 wells mapped, 89% were located within 30 minutes of a water testing facility, 15% were located within 0 to 10 minutes of a water testing facility, 48% were located between 10 and 20 minutes of a water testing facility and 37% were located within 20 to 30 minutes of a water testing facility. Further analysis revealed that access to water testing facilities may be influenced by the hours of operation of the facilities.

## Introduction

An estimated 238 000 to 450 000 people rely on private well water in Alberta.^1,2^ Water quality testing becomes an essential strategy for safeguarding public health among private well users. Recent studies found between 14.6% and 21% of wells in Alberta were contaminated with total coliform and up to 1.5% were contaminated with *E. coli*.^1,3^ Enteric viruses were also found in nearly 7% of well water samples.^4^ Due to the lack of routine water testing on many private water wells in Alberta, assessing well water contamination can be problematic.

Currently there is no mandatory legal requirement for private well owners in Alberta to routinely test their well water quality leaving the responsibility of well stewardship and management to well owners.^5^ Reported water testing rates among private well owners in Alberta are low. Approximately 11% of private well owners test their water annually, with only about 7% conducting a water test every 2 years.^2^ Recommendations emphasise the need to conduct microbiological water quality tests at least twice per year and chemical testing at least once every 3 years.^5^

Water testing is a preventative health behaviour that could help protect well users from health complications associated with drinking contaminated well water and an important component of well stewardship. Access to water testing services has been known to influence water testing behaviour.^6^ Accessibility to healthcare facilities is a barrier to healthcare delivery in Canada^7-10^ Decisions to seek health services may be influenced by quality of services offered in an area, distance and time to travel to health facilities, and the costs of accessing health services.^11^ Research suggests that access to preventative health facilities is an important factor influencing an individual’s decision to participate in preventative health behaviours^6,12,13^. A frequently cited barrier influencing water quality test submissions is the time inconvenience of submitting water samples which can be influenced by the proximity and availability of water testing services.^14-16^

Water quality submission policies and procedures in place may limit the accessibility and availability of public water testing services. Prior to the COVID-19 pandemic (March 2020), well water testing for microbiological contamination and chemical contamination was offered at no charge to well owners in Alberta through Alberta Health Services (AHS).^14^ Water sample acceptance time is restricted in water sample drop off locations (see Supplemental Appendix Table 1). Furthermore, current testing procedures for microbiological contaminants only consider samples submitted 24 hours after collection as viable.^5^ Subsequently, proximity to water testing facilities and the hours of operation (ie, water sample acceptance times) become important factors influencing water quality test submissions, especially if the hours of operation of these facilities are limited and inconvenient.^15,16^

Evaluating proximity to healthcare services has widely employed GIS tools. Network analysis in GIS offers a vector-based tool to solve routing problems based on road distance and travel times.^17^ Service areas are all streets that can be accessed within a specific travel time, in this study, drive time of a facility. Service area analysis has been used to evaluate the accessibility to healthcare facilities for emergency and acute inpatient services.^7,18^ However, literature on access to preventative healthcare services, specifically well water testing services is lacking. The objective of the study is to describe the drive times from water wells to public water testing facilities offered by AHS and describe availability based on the hours of operation of the facilities.

## Methods

### Data sources

Water well locations within the province were gathered from the Alberta Well Water Information Database (AWWID).^19^ AWWID contains data on over 400 000 wells drilled within the province since the early 1900′s. Water wells were selected based on the year in which the wells were drilled (ie, drill end date) between 2015 and 2021 as listed on the AWWID database. We selected domestic wells drilled within the last 6 years as it was more likely for them to currently be in use. Well selection was based on reported well use (ie, domestic) and classification as a new well. Duplicate well entries based on well identification numbers were eliminated.

Locations of AHS water testing facilities were gathered from the AHS website.^20^ Well water test facility coordinates were gathered from address locations input into Google maps. We assumed well owners thinking of conducting water testing would use Google maps (or a similar search engine) to locate the closest water testing facility. Data on the facility hours of operation were collected from the AHS water sample information page (see Supplemental Appendix Table 1). All shapefiles used in the development of maps were accessed through the University of Calgary’s Spatial and Numeric Data Services (SANDS).

### Data processing

**Figure 1.**
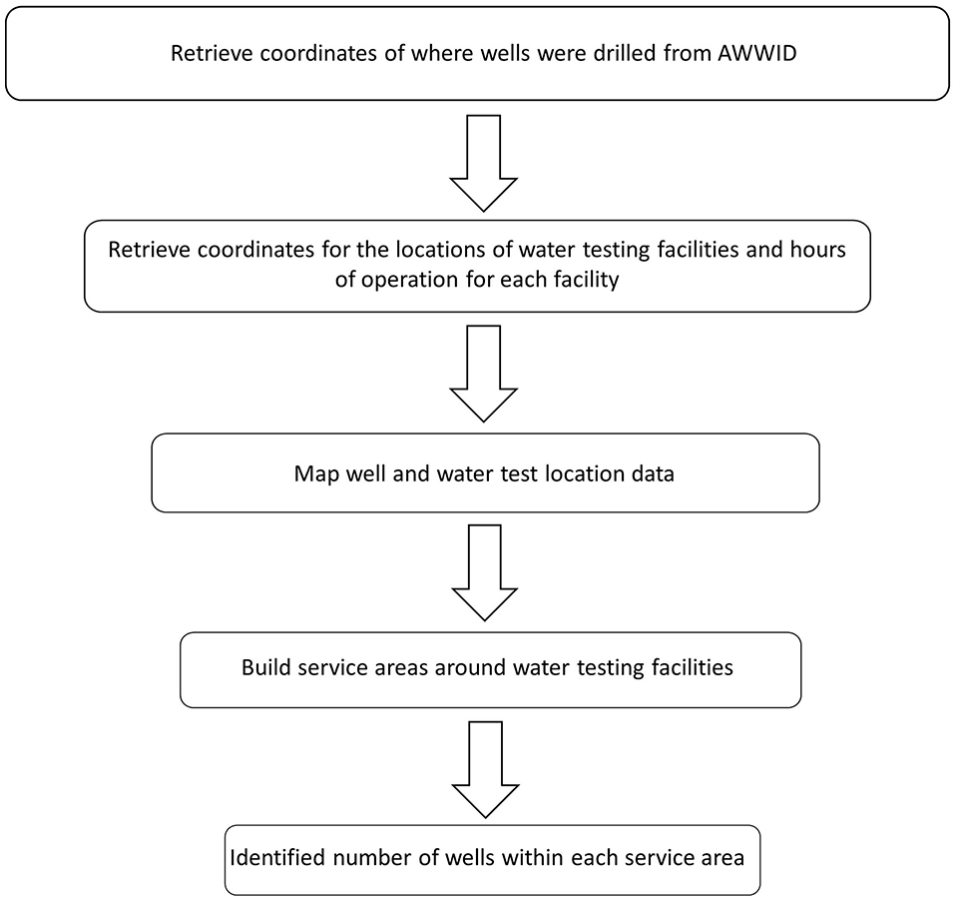
Workflow for determining number of wells within each service area.

Positional coordinates for well locations, water testing facilities and shapefiles for the Alberta Provincial boundary and Alberta Health Service regions were plotted in ArcGIS Pro. Data were projected into the NAD 1983 UTM Zone 11 coordinate system. Hours of operation (ie, day of the week and water sample pick up/drop off times) for the individual water testing facilities were added to each facility. To capture the variability in the operating hours of all the water testing facilities used in this analysis we selected three-time windows (ie, 3, 6 and 9 hours) as reference points to determine how many water wells would have access based on the hours of operation. The network analyst extension was used to build the service areas. Service area analyst tool was run using the water testing facility locations as facilities and well locations as incidences. Non-overlapping service area polygons were generated around each water testing facility based on travel times of 0 to 10, 10 to 20 and 20 to 30 minutes away from the facilities and a service area network dataset developed. We chose non-overlapping service areas built away from facilities as this creates individual polygons that are closest for each facility which would be similar to a routing option on a search engine like Google maps that would provide well owners with the closest water testing facility they can pick up and drop off sampling bottles. Although the utilisation of healthcare facilities will be different for sick role and preventive health behaviours,^21^ a 30-minute drive time has been used as an indicator of accessibility to health services.^22^ To get the number of wells within 3 drive times of increasing 10-minute intervals (ie, 0-10, 10-20 and 20-30 minutes), the selection tool was used, and wells were selected by location based on the drive time break to a water testing facility. The spatial join tool was used to create a layer of wells within each service area. The workflow for determining the number of wells within service areas of testing facilities is presented in Figure 1.

## Results

**Figure 2.**
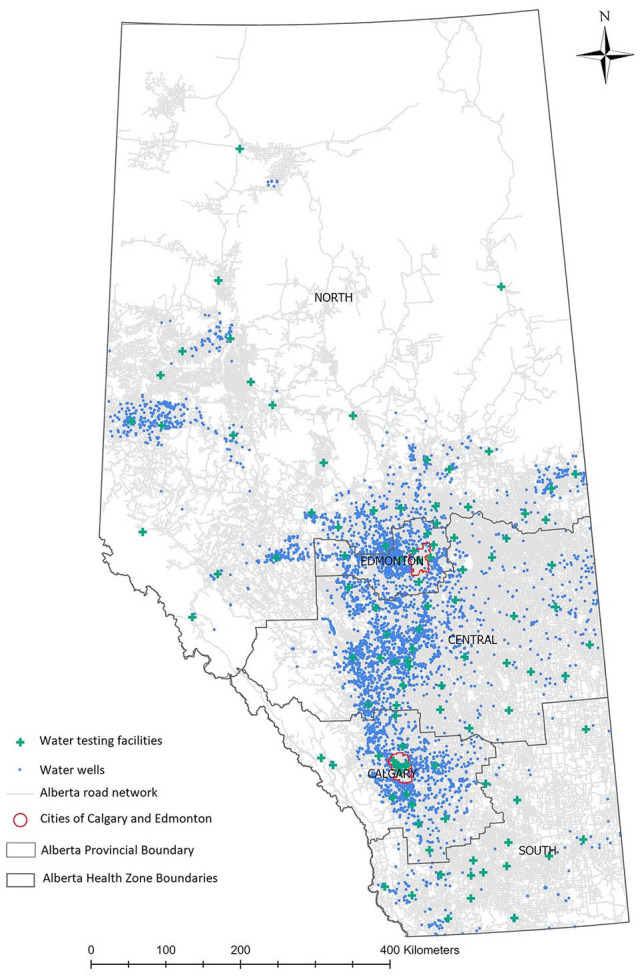
Water wells and water testing facility locations.

Well report data listed within the AWWID database numbered 426 451. Stratifying by well drill end date, well use, type of well and eliminating duplicated well ID entries resulted in a final sample of 5872 wells (Supplemental Appendix Table 2). Most water wells were located in the central and southern regions of the province in and around the Calgary-Edmonton corridor (Figure 2).

**Figure 3.**
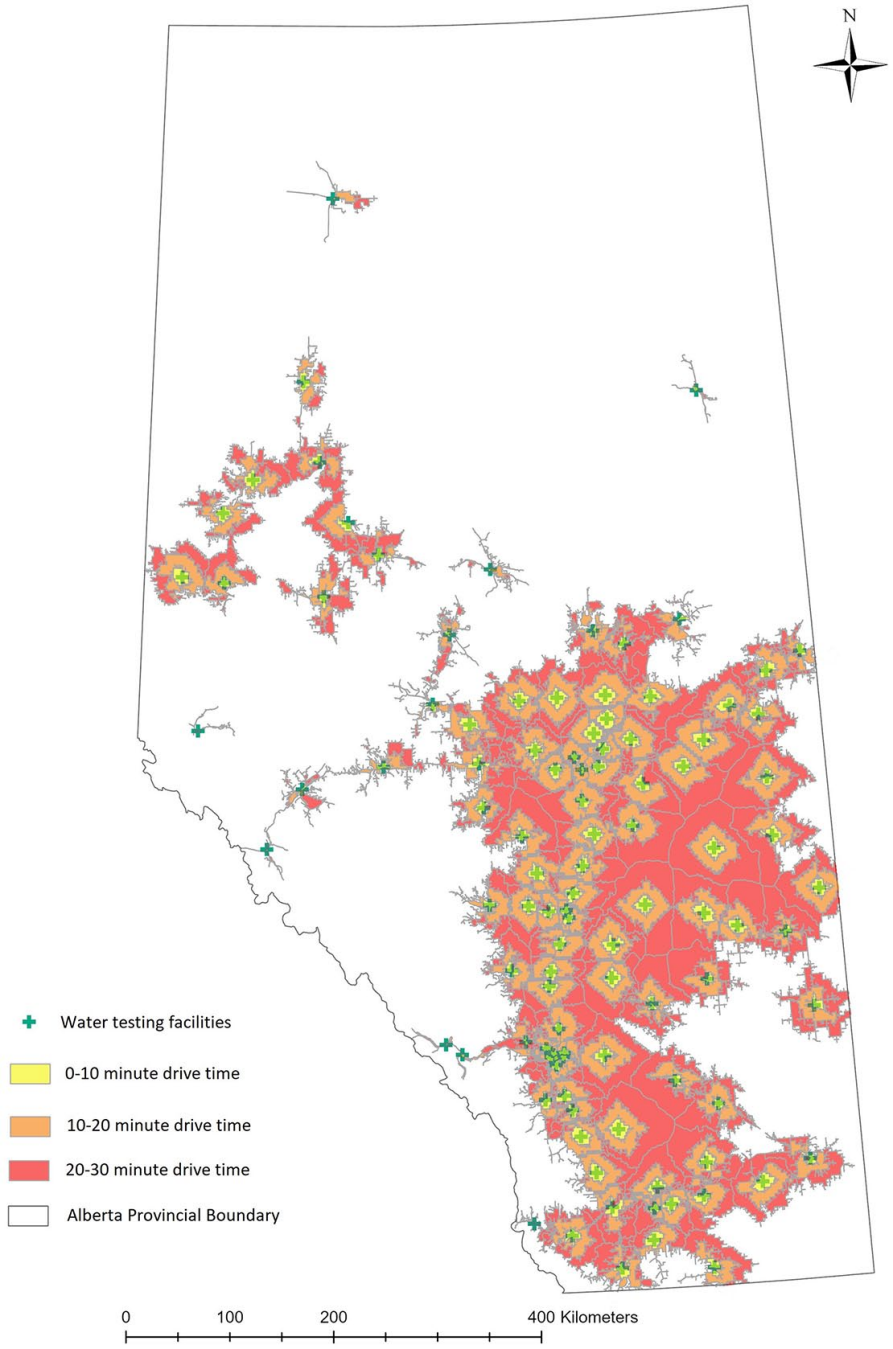
Service areas around water testing facilities.

A total of 107 AHS water testing facilities were identified and mapped (Figure 2). Non-overlapping service areas were developed around each AHS water testing facility based on 3 drive times (Figure 3). Eighty-nine percent (n = 5254) of the 5872 water wells geolocated were found within 30 minutes of a water testing facility: the numbers of water wells found within 0 to 10, 10 to 20 and 20 to 30 minutes of a water testing facility were 15% (n = 799), 48% (n = 2521) and 37% (n = 1934 wells), respectively. Of the 107 water testing facilities 48.7% (n = 52) of facilities were open on 1 day during the week, 17.8% (n = 19) of facilities were open on 2 days of the week, 16.8% (n = 18) of facilities were open on 3 days of the week. 6.6% (n = 7) of facilities were open 4 days of the week and 10.3% (n = 11) of facilities were open 5 days of the week. Mean (s.d.) hours of operation based on the number of days facilities allowed well owners to pick-up or drop off water sampling bottles was calculated. Facilities open 1 day a week were operated for 4.92 (1.81) hours. Facilities open 2 days operated for 4.5 (1.94) hours. Facilities open for 3 and 4 days in the week operated for 4.77 (1.77) and 5.43 (1.43) hours respectively. Facilities that opened on 5 days of the week had the shortest hours of operation 1.77 (1.53) hours. Mean (s.d.) hours of operation for all facilities was 4.53 (1.99).

**Figure 4.**
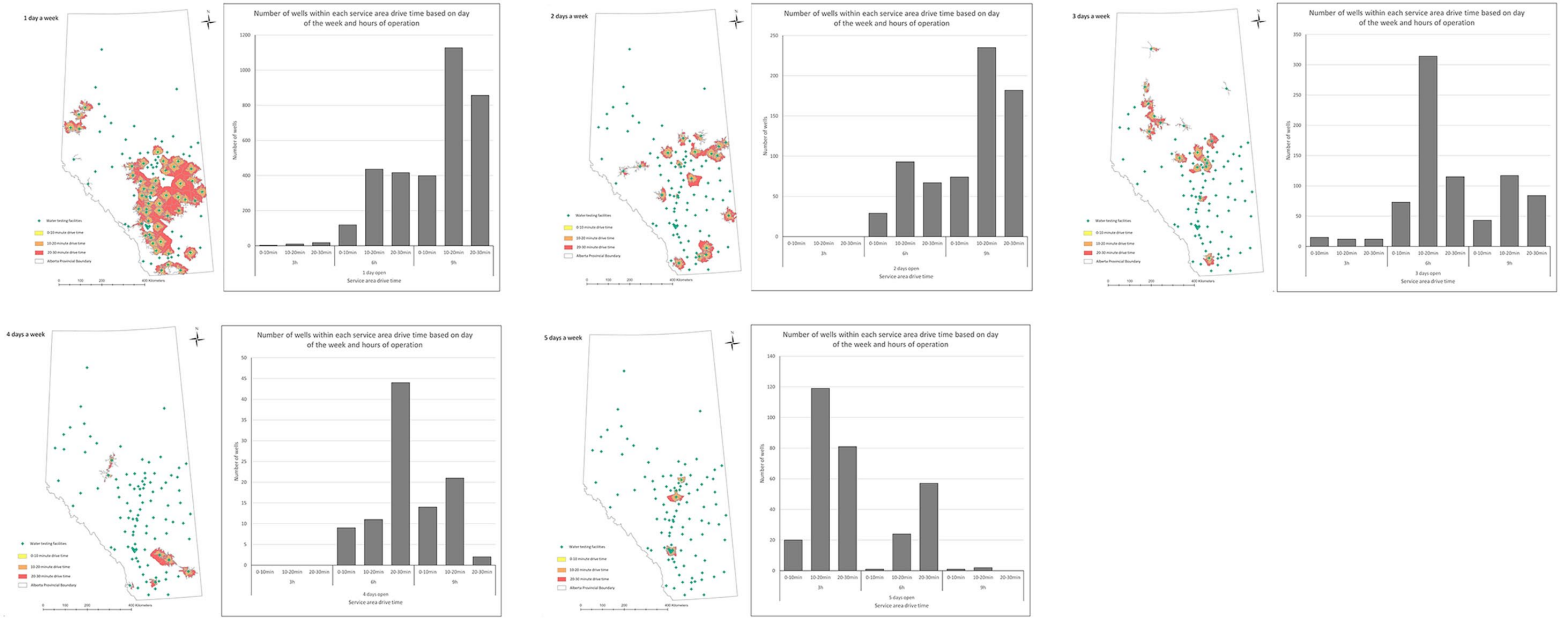
Service areas and number of wells with access to them based on the number of days/week open.

The number of wells present within each drive time break (ie, 0-10, 10-20 and 20-30 minutes) based on three-time windows (ie, 3, 6 and 9 hours) is presented in Figure 4.

The percentage of wells with access to water testing facilities varied by day of the week (see Table 1). The percentage of wells with access to water testing facilities based on hours of operation can be found in Table 2.

**Table 1.** Percentage of wells within 30 minutes of water testing based on number of days of the week facilities were open.

Percentage of wells within 30 min of testing facility	Number of facilities	Number of days per week open
64.00	52	1
12.90	19	2
14.90	18	3
1.92	7	4
5.80	11	5

**Table 2.** Percentage of wells with access to water testing facilities based on hours of operation.

Operating hours per day	3 h			6 h			9 h			Total
Time interval (min)	0-10	10-20	20-30	0-10	10-20	20-30	0-10	10-20	20-30	
% of wells 1 day*	0.03	0.19	0.34	2.24	8.29	7.91	7.59	22.02	16.29	64
% of wells 2 days	0	0	0	0.55	1.77	1.27	1.41	4.47	3.46	12.90
% of wells 3 days	0.28	0.23	0.23	1.39	5.98	2.18	0.82	2.23	1.60	14.90
% of wells 4 days	0	0	0	0.17	0.21	0.84	0.27	0.40	0.04	1.92
% of wells 5 days	0.38	2.26	1.54	0.02	0.46	1.08	0.02	0.04	0	5.80
% of wells all days	0.69	2.68	2.11	4.40	16.71	13.30	10.11	28.59	21.39	100

* Percentage of wells with access to water testing facilities as a proportion of the total number of wells in our sample within 30 minutes of a testing facility (ie, 5254) based on hours of operation.

Two-tailed difference in proportions tests were conducted to evaluate if there was a difference in the proportion of wells with access to testing facilities that were in close proximity (ie, 0-10 minutes) based on the hours of operation (ie, 3, 6 and 9 hours) and similarly if there was a difference in the proportion of wells with access to testing facilities that were further away (ie, 20-30 minutes) based on the service area polygons (Table 3).

**Table 3.** Difference in proportions of water wells with access to testing facilities based on hours of operation.

	Between 3 and 6 h				Between 3 and 9 h				Between 6 and 9 h			
	Close (0-10)		Far (20-30)		Close (0-10)		Far (20-30)		Close (0-10)		Far (20-30)	
% Wells	0.69	4.40	2.11	13.30	0.69	10.11	2.11	21.39	4.40	10.11	13.30	21.39
Z score	–0.13		–.0.15		–1.89		0.88		–3.8		2.17	
p value	0.89		0.88		0.06		0.37		0.00*		0.03*	

* Significant at *P* < .05.

## Discussion

A large proportion (89%) of water wells in our sample were within 30 minutes of AHS water testing facilities with 15%, 48% and 37% of water wells within 0 to 10, 10 to 20 and 20 to 30 minutes of AHS water testing facilities respectively. However, because well owners are required to pick up and drop off water sampling bottles for testing to be conducted, the drive times are potentially doubled. Our service areas were built assuming one-way travel from a well to a facility. The majority of well owners conducting water tests drive to and from testing facilities. Furthermore, as travel times in the service area analysis model were based on travelling at the maximum speed limit on each road, travel times from the well to water testing facilities may be longer than those predicted by service area buffers. This is because other factors along a road route (eg, stop signs, traffic lights and traffic flow) could increase travel times. Additional factors such as time of day, road surface and weather conditions could influence driving speeds, increasing expected travel times to water testing facilities.^7^

Buffers around each health facility assume equal access for individuals within buffer limits.^23^ However, access is determined in part by both availability and proximity and therefore may be limited by hours of operation of the health facility. Our study found the majority (52/107) of water testing facilities in Alberta were open on 1 day a week and their service areas captured 64% of the wells in our sample. This may have been due to the wide geographic spread of the facilities across more populated areas in the province. Facilities open on 2, 3, 4 and 5 days a week captured roughly 13%, 15%, 2% and 6% respectively of the wells within our sample (Table 1). Some of this variation may have been because some water testing facilities (eg, those open for 5 days a week) were in predominantly urban areas (ie, the city of Calgary or Edmonton) and therefore would expect fewer private water wells in areas with municipal water supplies. This would also explain why well testing facilities open for the most days in the week reported the fewest average hours of operation (ie, 1.77 hour) for submission of water tests; however, there were a few exceptions to this (ie, the provincial laboratories in Calgary and Edmonton) in which the microbiological tests are conducted. Once water samples are submitted to water testing facilities, they are couriered to the provincial labs in Calgary and Edmonton for microbiological analysis. Previous research in Alberta found that water quality test submissions tended to occur frequently mid-week (ie, Tuesdays and Wednesdays) as opposed to the end of the week or weekends when the water testing facilities were not open.^3^ This suggests the hours of operation of health facilities may influence how likely well users are to submit samples within the service area.

Based on hours of operation, our study used three-time windows to capture the variable hours of operation for all 107 water testing facilities. We found no significant difference between the proportion of wells that were close (ie, 0-10 minutes) and far (20-30 minutes) to water testing facilities between the 3 and 6-hours of operation time windows and 3 and 9-hour time windows. However, we did find a significant difference in the proportion of wells that were both close and far between the 6 and 9-hour time windows (Table 3). For facilities open on 1 and 2 days a week, longer hours of operation (ie, 6 hours or more) could increase availability of services to wells within their catchment area. For testing facilities that were open 3 and 4 days a week, we found the majority of wells had access within a 6-hour operating time. Facilities open 5 days a week had the most wells captured within a 3-hour operating time (Figure 4). Increasing hours of operation may have an impact on the availability of water testing services (ie, having more facilities open for longer hours in the day would give well owners a larger time window and more flexibility in submitting water tests).

## Conclusion and Future Directions

The objective of this study was to describe the proportion of wells within 3 estimated drive times of water testing facilities within Alberta. We found that 89% of wells within our sample were within 30 minutes of water testing facilities. Taking into consideration drive times would be doubled and road route variables are considered, the time taken to public water testing facilities may be a barrier to water sample submission and corroborates with previous literature on the inconvenience of water sample submissions.^15,16,24-26^ The use of GIS allows us to quantify travel times further exploring nuances in the access to water testing facilities. Using our methods, future research could utilise data on drive-times to water testing services as a predictor variable of water sample submission within different jurisdictions or catchments. This would be an advance on current methods that may be reliant on self-reported perceptions of inconvenience of water sample submissions. The use of service area analysis and assessment of drive times can be applied to predict what section of the population are vulnerable and less likely to seek out preventative health services such as water testing.^27^

## Limitations

To the best of our knowledge, our research is the first study to quantify and describe drive times and hours of operation of well water testing services. Despite the applicability of our study to informing future well water testing studies, there were some limitations. The positional accuracy of some well locations may have not been exact and limited to the quarter section the well was drilled in. Furthermore, although, our inclusion criteria for wells by type of well, well use and well age tried to eliminate the possibility of having inactive wells in our final sample, some wells within the database may have been inactive. Our study was also limited as we did not have access to provincial well water quality testing data. Rural residents may also travel considerable distances for work and shopping, and the assessment of drive times may not be the ideal way of assessing whether trips to water testing facilities are burdensome. We only described the proximity of water wells to public health water testing facilities in Alberta. These results may not be generalisable to other provinces and territories in Canada or apply to well owners who choose to test their water through privately owned water testing facilities. Very recent changes to hours of operation and costs of testing at the time of writing of this article may influence water testing behaviour. Finally, although accessibility to water testing services is important, there may be other factors that influence water quality test submissions including sensory perceptions of water quality, lack of knowledge about water testing and well stewardship, cost of testing, use of water treatment, and perceived risk of well water contamination.^6,14,28-30^

## Supplemental Material

sj-xlsx-1-ehi-10.1177_11786302221137437 – Supplemental material for Proximity of Water Wells to Public Water Testing Facilities in Alberta Using Drive Times Click here for additional data file. Supplemental material, sj-xlsx-1-ehi-10.1177_11786302221137437 for Proximity of Water Wells to Public Water Testing Facilities in Alberta Using Drive Times by Abraham Munene and David C. Hall in Environmental Health Insights
